# Microcirculatory Monitoring in Children with Congenital Heart Disease Before and After Cardiac Surgery

**DOI:** 10.1007/s12265-023-10407-4

**Published:** 2023-07-14

**Authors:** Özge Erdem, Jurgen C. de Graaff, Matthias P. Hilty, Ulrike S. Kraemer, Inge I. de Liefde, Joost van Rosmalen, Can Ince, Dick Tibboel, Jan Willem Kuiper

**Affiliations:** 1 Intensive Care and department of Pediatric Surgery, Erasmus MC University Medical Center–Sophia Children’s Hospital, Rotterdam, The Netherlands; 2 Department of Anesthesiology, Erasmus MC University Medical Center, Rotterdam, The Netherlands; 3 Institute of Intensive Care Medicine, University Hospital of Zurich, Zurich, Switzerland; 4 Department of Biostatistics, Erasmus MC University Medical Center, Rotterdam, The Netherlands; 5 Department of Epidemiology, Erasmus MC University Medical Center, Rotterdam, The Netherlands; 6 Department of Intensive Care, Erasmus MC University Medical Center, Rotterdam, The Netherlands

**Keywords:** Cardiopulmonary bypass, Congenital heart disease, Microcirculation, Hemodynamic monitoring, Cardiac surgery

## Abstract

In this prospective observational study, we investigated whether congenital heart disease (CHD) affects the microcirculation and whether the microcirculation is altered following cardiac surgery with cardiopulmonary bypass (CPB). Thirty-eight children with CHD undergoing cardiac surgery with CPB and 35 children undergoing elective, non-cardiac surgery were included. Repeated non-invasive sublingual microcirculatory measurements were performed with handheld vital microscopy. Before surgery, children with CHD showed similar perfused vessel densities and red blood cell velocities (RBCv) but less perfused vessels (*p* < 0.001), lower perfusion quality (*p* < 0.001), and higher small vessel densities (*p* = 0.039) than children without CHD. After cardiac surgery, perfused vessel densities and perfusion quality of small vessels declined (*p* = 0.025 and *p* = 0.032), while RBCv increased (*p* = 0.032). We demonstrated that CHD was associated with decreased microcirculatory perfusion and increased capillary recruitment. The microcirculation was further impaired after cardiac surgery. Decreased microcirculatory perfusion could be a warning sign for altered tissue oxygenation and requires further exploration.

**Graphical abstract:**

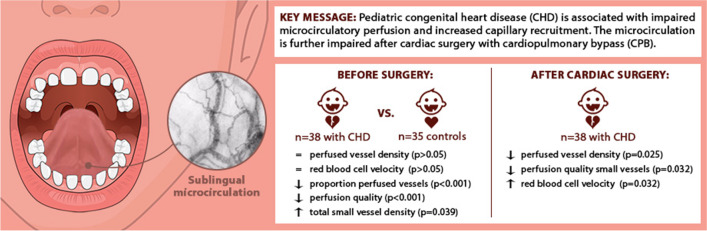

**Supplementary Information:**

The online version contains supplementary material available at 10.1007/s12265-023-10407-4.

## Introduction

Congenital heart disease (CHD) is the most common congenital anomaly, affecting an average 8.0 per 1000 live births [1]. CHD, depending on the anomaly, affects the circulatory system and subsequently the oxygen transport to tissues. Oxygen transport is regulated by the microcirculation. In clinical practice, we have little insight into how CHD and surgical repair of the anomaly with cardiopulmonary bypass (CPB) affects the microcirculation. Using handheld vital microscopy, the microcirculation can now be visualized in a non-invasive manner at patients’ bedside [2]. With the introduction of automated analysis software MicroTools (Active Medical B.V., Leiden, Netherlands), red blood cell velocity (RBCv) can be assessed as an additional microcirculatory parameter [3]. This offers further insight into microcirculatory function.

One pediatric study looked into the effect of CHD on the microcirculation [4]. This study found that patients with cyanotic CHD showed higher vessel densities than those with non-cyanotic CHD but the study lacked a control group without CHD. Three pediatric studies on the effect of cardiac surgery with CPB on the microcirculation have shown conflicting results. A study into the cutaneous microcirculation showed decreased vessel densities and perfusion after surgery [5]. Another study showed that the sublingual microcirculation was unaltered after surgery [6]. However, patients with cyanotic CHD showed different time trends for microcirculatory parameters after surgery than those with non-cyanotic CHD. A recent study showed that the sublingual microcirculation was impaired intraoperatively but recovered to baseline values after surgery [7].

In this study, we assessed the sublingual microcirculation perioperatively with handheld vital microscopy in children with CHD undergoing cardiac surgery with CPB. We hypothesized that the microcirculation of children with CHD is different from that of children without CHD before surgery, and the microcirculation of children with CHD is altered in the first hours following surgery.

## Methods

### Design, Setting, and Study Population

We performed a single-center prospective observational cohort study in a tertiary university children’s hospital over a 3-year period. Children until 18 years old with CHD undergoing elective cardiac surgery with CPB were eligible for inclusion in the cardiac surgery group. Children until 18 years old undergoing elective non-cardiac surgery with the ASA Physical Status Classification System in either ASA I or II and inhalation induction of anesthetics were eligible for inclusion in the non-cardiac surgery group. In both groups, patients were excluded if malformations of the sublingual area were present. Patients were also excluded in the non-cardiac surgery group in case of prematurity; cardiovascular, renal, or oncological disease, or genetic syndromes. Due to a lack of neonates undergoing elective non-cardiac surgery, exact age-matching was not possible. Written informed consent was obtained before enrollment. The study was approved by the medical ethical review board of the Erasmus University Medical Center (MEC2011-400; MEC2017-542). All procedures followed were in accordance with the ethical standards of the responsible committee on human experimentation (institutional and national) and with the Helsinki Declaration of 1975, as revised in 2000.

### Data Collection

Patient characteristics, routine hemodynamic and laboratory parameters, and administered drugs were obtained from electronic medical records. The Risk Adjustment for Congenital Heart Surgery (RACHS-1) score was calculated to assess the risk-adjusted in-hospital mortality of CHD [8]. In this cohort, the following CHD were considered cyanotic: cyanotic tetralogy of Fallot, total anomalous pulmonary venous connection, hypoplastic left heart syndrome, transposition of the great arteries, truncus arteriosus, tricuspid atresia, pulmonary atresia, and critical pulmonary stenosis. The inotrope score (IS) and the vasoactive-inotrope score (VIS) were calculated to assess the severity of inotropic and vasoactive drug support [9].

### Anesthetic Procedures and Cardiopulmonary Bypass

In the cardiac surgery group, hospital protocols were followed for both anesthetic and surgical procedures. Induction of anesthesia was achieved either through inhalation gas sevoflurane (between 6 and 8%) or administration of propofol (1–5 mg/kg) followed by administration of midazolam (0.1–0.6 mg/kg), sufentanil (0.1–1.0 μg/kg), and pancuronium (0.1–0.3 mg/kg) or rocuronium (0.3–1.2 mg/kg). Patients were intubated with a cuffed endotracheal tube and received pressure-controlled ventilation. Maintenance of anesthesia was achieved through continuous administration of either midazolam (200–300 μg/kg/h) or propofol (3–8 mg/kg/h) and either sufentanil (1–5 μg/kg/h) or remifentanil (0.1–2 μg/kg/min). Patients also received cefazolin (50 mg/kg) and, for any surgery except closure of atrial or ventricular septal defect, methylprednisolone (30 mg/kg). CPB was performed using non-pulsatile flow. Anticoagulation was achieved through the administration of unfractionated heparin (an initial dose of 300 IU/kg body weight followed by an additional dose of 500–2500 IU depending on the activated clotting time and patient’s body surface). To antagonize anticoagulation after CPB, protamine was administered.

In the non-cardiac surgery group, a study protocol was followed for anesthetic procedures. Induction of anesthesia was achieved through inhalation gas sevoflurane (inspiratory sevoflurane between 6 and 8% in 60–80% oxygen), followed by administration of either fentanyl (1.0–5.0 μg/kg) or sufentanil (0.1–0.5 μg/kg) and rocuronium (0.5–1.0 mg/kg). Patients were then also intubated with a cuffed endotracheal tube and received pressure-controlled ventilation and received anesthesia maintenance with sevoflurane, striving for end-tidal sevoflurane concentration between 2 and 3%.

### Microcirculatory Monitoring

Microcirculatory monitoring was performed with the CytoCam (Braedius Medical, Huizen, Netherlands), a handheld vital microscope with incident dark field illumination (IDF) [10]. International consensus guidelines were followed for assessment and analysis [11, 12]. Five 6-seconds clips were obtained by one observer from the sublingual mucosa at the following time points in the cardiac surgery group: (T_1_) before surgery (after induction of anesthetics and start of mechanical ventilation), (T_2_) after closure of the surgical wound, (T_3_) 1 h after surgery, (T_4_) 4 h after surgery, and (T_5_) 6 h after surgery. The non-cardiac surgery group was only measured at (T_1_)*.* Microcirculation data was not used for clinical decision-making.

After completing each group, clips were analyzed offline and coded [13]. The three of five clips per time point with the best quality were used for analysis. The selected clips were analyzed using analysis software AVA tools 3.2 (MicroVision Medical, Amsterdam, Netherlands) and MicroTools [3]. To estimate the oxygen-extraction capacity of the microcirculation, total vessel density (TVD, mm/mm^2^), proportion of perfused vessels (PPV, %), and perfused vessel density (PVD, mm/mm^2^) were assessed with AVA tools [11]. TVD is the density of the measured area covered by vessels. PPV is the proportion of well-perfused vessels. Multiplication of TVD and PPV gives the PVD. To estimate the oxygen transport capacity of the microcirculation, microcirculatory flow index (MFI) and RBCv were assessed with AVA tools and MicroTools, respectively. MFI is a qualitative score ranging from 0 (no flow) to 3 (normal flow*)*. An MFI < 2.6 was defined as disturbed perfusion quality [14]. RBCv is the weighted mean velocity of the absolute RBCv in all small vessels within the field of view [3]. For all parameters except RBCv, a distinction was made between all vessels with a diameter < 100 μm and small vessels with a diameter < 20 μm (mostly capillaries), as indicated by the subscript. Parameters without subscript refer to both distinctions.

### Statistical Analysis

Continuous data are presented as median (interquartile range) and categorical data as frequency (%). To compare the cardiac surgery group and the non-cardiac surgery group at (T_1_) and the secondary subgroup analysis to compare cyanotic CHD and non-cyanotic CHD at (T_1_), the Mann-Whitney *U* test was performed for continuous data and the Pearson’s chi-squared test for categorical data. The Spearman rank correlation coefficient was calculated to assess correlations. Previous research showed that age and sex affect vessel density in young children [15–17]. Therefore, linear regression models were built for TVD and PVD at (T_1_) with covariate group (cardiac surgery group or non-cardiac surgery group), age, and sex. To compare the cardiac surgery group between (T_1_) and (T_2_), the Wilcoxon signed-rank test was performed for continuous parameters. For secondary analyses, linear mixed models were built for TVD and PVD with covariate time point, age, and sex. To explore whether TVD and PVD changed from (T_1_) through (T_5_) in the cardiac surgery group while accounting for repeated measures, linear mixed models were built with time point (T_1_ to T_5_), age, and sex as independent variables. To account for the within-subject correlations, a random intercept was included in these models. To explore whether MFI changed from (T_1_) to (T_5_), a generalized estimating equation logistic regression model was built with time point as the only independent variable and MFI < 2.6 as the dichotomous dependent variable. No imputation of missing values was performed. For all analyses, two-sided *p*-values < 0.05 were considered statistically significant. IBM SPSS statistics 24 (IBM, Armonk, NY, USA) was used for all statistical analyses. The sample size was calculated using a power analysis for the difference in PVD between two time points and between two groups. No data on effect sizes were available. With a two-sided *α* of 0.05 and a desired power of 80%, 34 subjects undergoing elective cardiac surgery were needed to detect an expected difference of 0.6 SDs between two time points for PVD, corresponding with a medium effect size. With a two-sided *α* of 0.05 and a desired power of 80%, 34 subjects undergoing elective non-cardiac surgery were needed to detect an expected difference of 0.7 SDs between the two groups, corresponding with a medium to large effect size.

## Results

### Demographics of the Study Population

**Fig. 1:**
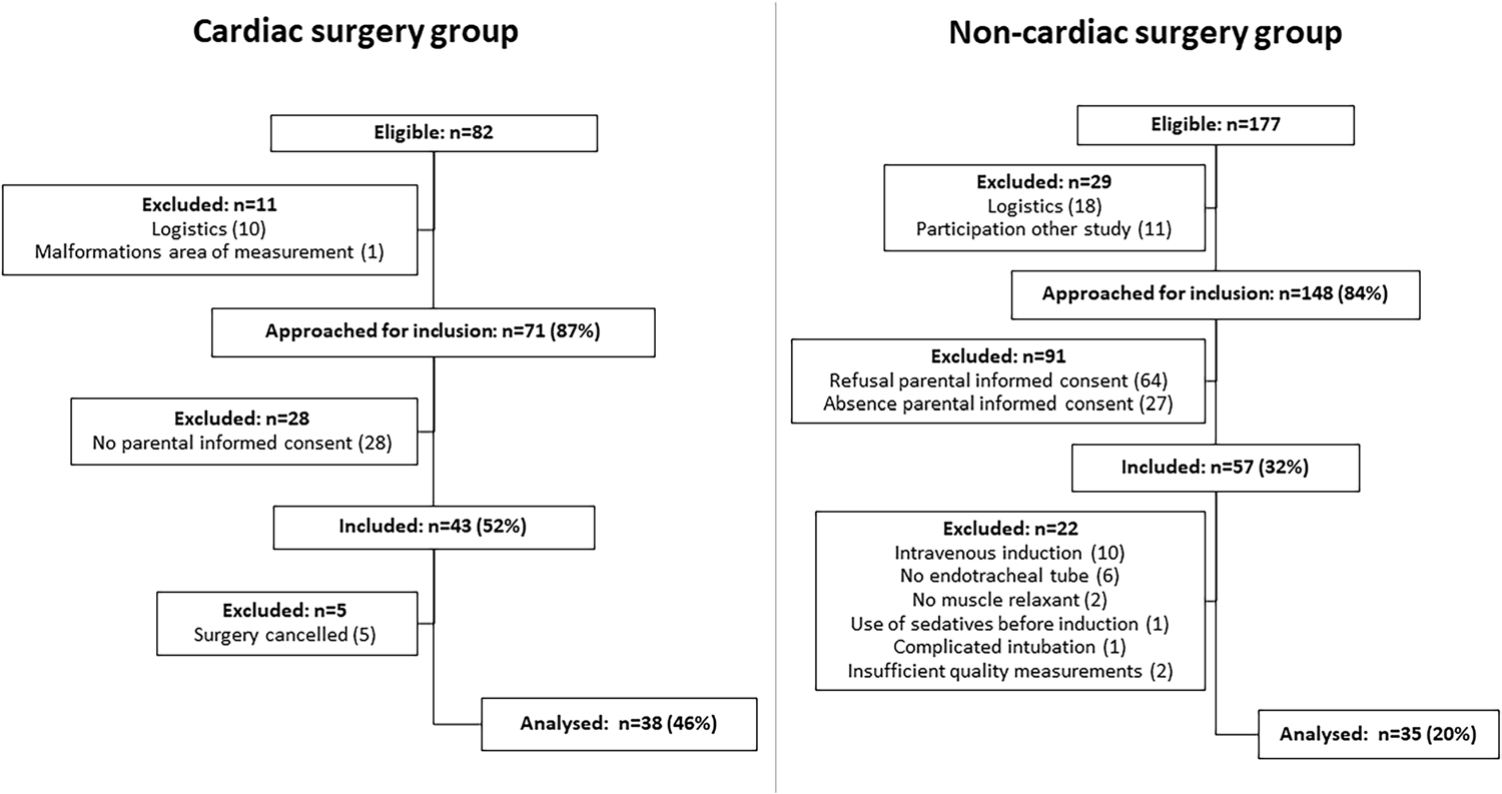
Flow chart inclusion of the study groups

Thirty-eight children with CHD and 35 children without were enrolled in the study. Figure 1 shows the flow charts for inclusion. Table 1 shows the demographics. Online Resource 1 Tables 1 and 2 show the diagnoses and the surgeries. The median age of the cardiac surgery group was 0.6 years (0.3–3.4). The median age of the non-cardiac surgery group was 1.3 years (0.6–3.6). The two groups did not significantly differ in sex and age.

**Table 1:** Demographics and vital signs at (T_1_) before surgery

Variable	Cardiac surgery group	Non-cardiac surgery group	*p*-value
Patients, *n*	38	35	-
Female, *n* (%)	16 (42%)	15 (43%)	0.810
Age, years	0.62 (0.31–3.37)	1.29 (0.58–3.57)	0.085
Neonate, *n* (%)	5 (13%)	0 (0%)	0.027*
Genetic disorders, *n* (%)	8 (21%)	0 (0%)	< 0.001*
Cyanotic heart defect, *n* (%)	10 (26%)	-	-
ASA physical status classification			
I	0	25 (71%)	< 0.001*
II	8 (21%)	10 (29%)	
III	28 (74%)	0	
IV	2 (5%)	0	
Mean arterial pressure, mmHg	56 (48–63)^a^	59 (56–64)^b^	0.096
Heart rate, bpm	122 (103–129)	125 (118–138)	0.027*
Peripheral oxygen saturation, %	97 (92–100)	100 (99–100)	< 0.001*
Fraction inspired oxygen, %	35 (28–50)	43 (40–66)	0.003*
End tidal CO2, kPa	5.2 (4.9–6.3)	5.7 (5.4–5.8)	0.847
Core body temperature, °C	36.4 (35.5–36.7)	36.7 (36.4–36.9)	0.029*

Data from (T_1_) before surgery are shown. Continuous data are presented as median (interquartile range) and categorical data as *n* (%).The last column shows the *p*-values based on the Mann-Whitney *U* test for continuous data and the Pearson chi-square test for categorical data. *ASA* American Society of Anesthesiologists

*A *p*-value < 0.05 was considered significant

^a^Assessed with an arterial catheter

^b^Assessed with non-invasive blood pressure measurements

Nine patients (24%) had cyanotic CHD (6 with single ventricular physiology, 2 with transposition of the great arteries, 1 with tetralogy of Fallot). These patients had higher median hemoglobin levels than those with non-cyanotic CHD (8.7 mmol/L vs. 7.0 mmol/L; *p* = 0.002) and higher median hematocrit levels (0.43L/L vs. 0.35L/L; *p* = 0.002) at (T_1_). These patients had lower SaO_2_ levels (93% vs. 100%; *p* = 0.004) and SpO_2_ levels (91% vs. 98%; *p* = 0.003).

Successful microcirculatory measurements could be obtained in 35 of 38 CHD patients, shown in Table 2. Data of 3 patients at (T_1_) and 4 at (T_2_) were of insufficient quality for analysis. Seven patients at (T_3_), 17 at (T_4_), and 31 at (T_5_) could not be measured because they were extubated and no longer sedated. The missing data from (T_3_) and onwards were seen as missing not at random.

**Table 2:** Perioperative demographics of the cardiac surgery group

	Time point					
Variable	(T_1_) Before surgery	(T_2_) After wound closure	*p*-value	(T_3_) 1 h after surgery	(T_4_) 4 h after surgery	(T_5_) 6 h after surgery
Patients, *n*	38	38	-	38	38	38
Microcirculatory measurements, *n* (%)	35 (92%)	34 (89%)	-	31 (82%)	21 (55%)	7 (18%)
Intubated and sedated, *n* (%)	38 (100%)	38 (100%)	-	31 (82%)	21 (55%)	7 (18%)
Mean arterial pressure, mmHg	56 (48–63)	62 (52–65)	0.051	66 (60–71)	64 (54–68)	58 (47–62)
Heart rate, bpm	122 (103–129)	154 (129–170)	< 0.001*	147 (125–160)	154 (143–167)	155 (150–158)
Central venous pressure, mmHg	8 (4–11)	12 (8–16)	0.001*	11 (8–13)	11 (9–13)	15 (10–18)
Peripheral oxygen saturation, %	97 (92–100)	99 (97–100)	0.086	99 (98–100)	98 (95–99)	95 (95–95)
Core body temperature, °C	36.4 (35.5–36.7)	36.6 (36.0–37.1)	0.034*	36.9 (36.3–37.2)	37.3 (37.0–37.8)	37.1 (36.5–37.4)
VIS	0	3.0 (1.1–5.0)	< 0.001*	3.0 (0.5–5.0)	3.1 (1.0–5.0)	4.9 (3.0–7.0)
IS	0	4.0 (1.5–8.0)	< 0.001*	3.0 (0.7–5.0)	3.1 (1.0–5.0)	5.7 (4.8–13.0)
Hemoglobin, mmol/L	7.1 (6.3–8.2)	6.2 (5.9–7.0)	0.030*	7.4 (6.6–7.9)	7.9 (7.0–8.5)	8.7 (7.7–9.5)
Hematocrit, L/L	0.35 (0.31–0.41)	0.31 (0.29–0.35)	0.047*	0.33 (0.28–0.38)	0.39 (0.33–0.42)	-
Lactate, mmol/L	0.6 (0.5–0.8)	1.1 (0.9–1.6)	< 0.001*	1.0 (0.8–1.3)	1 (0.8–1.5)	1.6 (1.0–2.2)

Continuous data are presented as median (interquartile range) and categorical data as *n* (%). The 4th column shows the *p*-values based on the Wilcoxon signed-rank test for continuous data to compare (T_1_) before surgery and (T_2_) after closure of the surgical wound. *CHD* congenital heart disease, *IS* inotrope score, *VIS* vasoactive inotropic score

*A *p*-value < 0.05 was considered significant

### The Effect of CHD on the Microcirculation

**Fig. 2:**
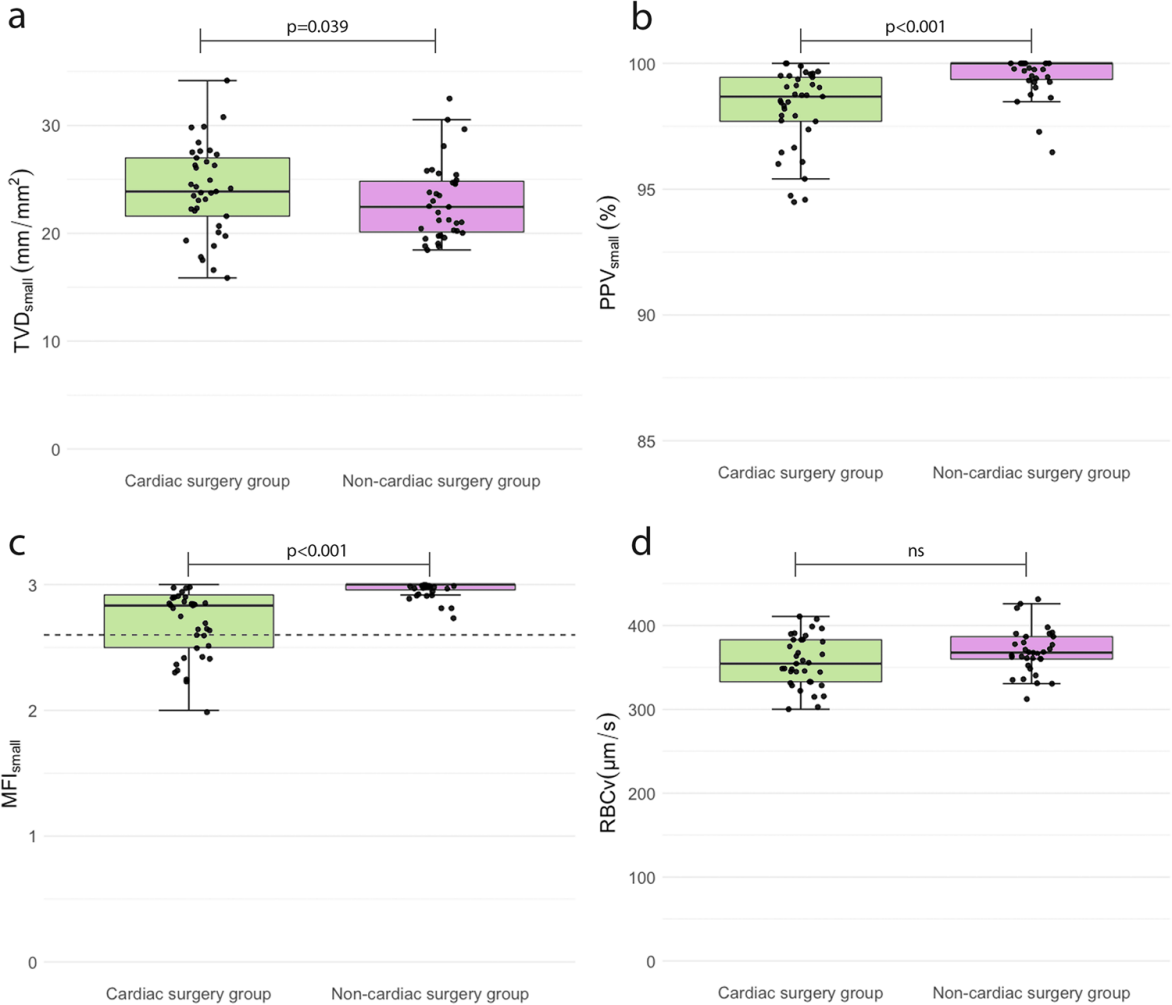
Microcirculatory parameters at (T1) before surgery: cardiac surgery group versus non-cardiac surgery group. The box plots are median data (interquartile range) of microcirculatory parameters for small vessels (< 20 μm) at (T_1_) before surgery. The dotted line in graph **c** represents the cutt-off value of 2.6 for a disturbed MFI. The *p*-value for TVD_small_ was derived from the linear regression model, corrected for age and sex. The *p*-values for PPV_small_, MFI_small_, and RBCv were derived from the Mann-Whitney U tests. A *p*-value < 0.05 was considered significant. NS = *p*-value was not significant. TVD total vessel density, PPV proportion of perfused vessels, MFI microcirculatory flow index, *RBCv* red blood cell velocity

At (T_1_) *before surgery*, TVD, PVD, and RBCv did not significantly differ between the cardiac surgery and the non-cardiac surgery group (Fig. 2 and Online Resource 1 table 3). PPV and MFI were significantly lower in the cardiac surgery group (*p* < 0.001). Age was correlated with TVD_all_ (*ρ* = − 0.500, *p* < 0.001), TVD_small_ (*ρ *= −0.471, *p *< 0.001), PVD_all_ (*ρ* = − 0.593, *p* < 0.001), and PVD_small_ (ρ = 0.578, p < 0.001) at (T_1_). Corrected for age and sex, TVD_small_ was significantly higher at (T_1_) in the cardiac surgery group than in the non-cardiac surgery group (*p* = 0.039) (Fig. 2 and Online Resource 1 table 4). Microcirculatory parameters of patients with a cyanotic CHD did not significantly differ from those with a non-cyanotic CHD at (T_1_) (data not shown).

Thus, at (T_1_) *before surgery,* the cardiac surgery group showed significantly lower PPV and MFI but higher TVD_small_ than the non-cardiac surgery group. RBCv, TVD_all_, and PVD did not differ between the groups.

### Microcirculatory Changes After Cardiac Surgery

Online Resource 1 table 5 shows the perioperative demographics. The median duration of cardiac surgery was 173 min. The median duration of CPB was 101 min. During 35 of the 38 surgeries, aortic cross-clamping was applied for a median duration of 32 min. Deep hypothermic circulatory arrest and antegrade cerebral perfusion were applied during 3 surgeries. The median ICU duration of stay was 1 day while the median duration of hospital stay was 7 days. All patients survived. Table 2 shows the systemic hemodynamic and laboratory parameters of the cardiac surgery group over time. Comparing (T_1_) and (T_2_), heart rates, central venous pressures, body temperatures, and serum lactate levels were significantly higher after surgery. Hemoglobin and hematocrit levels were significantly lower after surgery.

**Fig. 3:**
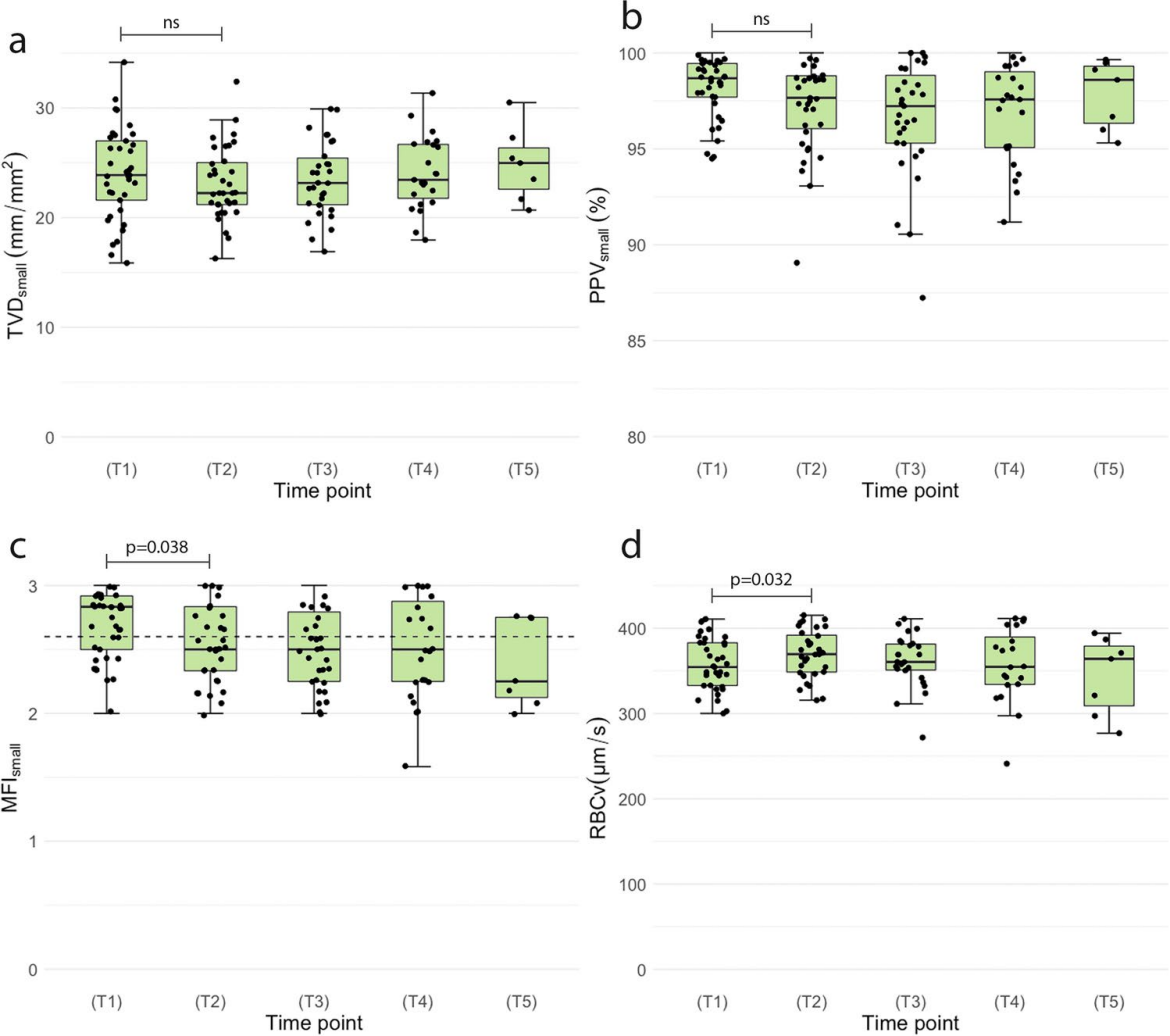
Microcirculatory parameters of the cardiac surgery group over time. The box plots are median data (interquartile range) of microcirculatory parameters for small vessels (< 20 μm) at the following time points: (T_1_) before surgery, (T_2_) after wound closure, (T_3_) 1 h after surgery, (T_4_) 4 h after surgery, and (T_5_) 6 h after surgery. The black dots represent the single data points. The dotted line of graph **c** represents the cut-off value of 2.6 for a disturbed MFI. The *p*-value for TVD_small_ was derived from the linear mixed model to compare (T_1_) versus (T_2_), corrected for age and sex. The Wilcoxon signed-rank test was used to compare (T_1_) versus (T_2_) for MFI_smal_, PPV_small_, and RBCv. A *p*-value < 0.05 was considered significant. NS = *p*-value was not significant. TVD total vessel density, PPV proportion of perfused vessels, MFI microcirculatory flow index, RBCv red blood cell velocity

At (T_2_) after wound closure, MFI_small_ was significantly lower than at (T_1_) (*p* = 0.038), while RBCv was significantly higher (*p* = 0.032), shown in Fig. 3 and Online Resource 1 table 6. Other parameters did not significantly differ between (T_1_) and (T_2_). Corrected for age and sex, TVD_all_ and PVD_all_ were significantly lower at (T_2_) than at (T_1_) (*p* = 0.032 and *p* = 0.025, respectively) (Online Resource 1 table 7). The probabilities of having a disturbed MFI_all_ and MFI_small_ increased after surgery (*p* = 0.041 and *p* = 0.008, respectively). Corrected for age and sex, TVD and PVD did not significantly differ over time (data not shown).

In summary, the cardiac surgery group showed lower MFI_small_, TVD_all_, and PVD_all_ but higher RBCv after surgery with CPB. The probability of having a disturbed MFI was higher after surgery.

## Discussion

Our study demonstrated that the sublingual microcirculation of children with CHD differs from that of children without CHD before surgery and was altered in the first hours following cardiac surgery with CPB.

### The Effect of CHD on the Microcirculation

Our study showed that the sublingual microcirculation of children with CHD showed less perfused vessels, lower perfusion quality, and higher small vessel densities than children without CHD but similar RBCv. Whether these statistical differences have actual clinical implications is unclear because reference values for microcirculatory parameters are lacking. However, the decreased perfusion could have resulted from the presence of CHD and could affect oxygen delivery. The increased small vessel density could be a sign of capillary recruitment to expand the oxygen-extraction capacity, to compensate for the decreased perfusion.

Secondary analyses also showed that children with cyanotic CHD had a similar microcirculation as those with a non-cyanotic CHD, while they did exhibit higher hemoglobin and hematocrit levels. This is contrary to findings by González et al., who showed cyanotic patients to have higher vessel densities, a possible compensatory mechanism for chronic hypoxemia [4, 7]. The difference between our findings could have resulted from the classification of cyanotic CHD. With the small number of study patients with cyanotic CHD (*n* = 7), we also lacked power to perform analyses to correct for hemoglobin or hematocrit levels.

### Microcirculatory Changes After Cardiac Surgery

Our study showed that the sublingual microcirculation of children with CHD was altered in the first hours following cardiac surgery with CPB. While the perfusion quality of small vessels and large vessel densities decreased directly after surgery, RBCv increased. The probability of having a disturbed perfusion quality of small vessels was also higher in the first hours after surgery than before surgery.

Our findings support existing adult literature showing a generalized disturbed microcirculation after cardiac surgery with CPB. Several studies showed that the onset of CPB induced decreased microcirculatory perfusion and density and these disturbances persisted through the early postoperative period [18–24].

Contrary to adult literature, limited and conflicting information is available on how cardiac surgery with CPB affects the pediatric microcirculation. Nussbaum et al. studied the transcutaneous microcirculation, a non-validated location for measurements, with a previous generation microscope, sidestream dark field imaging (SDF). They found decreased perfusion and perfusion quality after cardiac surgery [5]. Our study showed similar perfusion changes in the sublingual microcirculation. However, they measured the microcirculation the day before surgery instead of after induction of anesthesia. Scolletta et al. studied the sublingual microcirculation, also with SDF, but did not find microcirculatory changes after surgery. They did find that patients with cyanotic CHD showed different time trends for TVD and PPV [6]. Due to our small number of patients with cyanotic CHD and missing data, we could not replicate these findings. González Cortés et al. showed similar postoperative results with SDF in the sublingual microcirculation [7]. Microcirculatory parameters worsened intraoperatively and microcirculatory parameters returned to baseline values postoperatively. There are several explanations for our different results. We used IDF, a newer technique proven to assess higher quality imaging and detect more vessels than SDF [25]. The postoperative evaluation in the study of González Cortés et al. took place at a single time point before planned extubation before transfer to the PICU. We measured patients at multiple fixed time points. TVD and PVD were also corrected for age in our study, as previous studies showed age to affect microcirculatory parameters [15–17].

Decreased perfusion and vessel density after surgery could have resulted from decreased myocardial function, due to ischemic and reperfusion injury following cardiac surgery with CPB [26]. An additional reason would be the local endothelial vessel damage caused by reperfusion and systemic inflammation following CPB [27, 28]. Previous studies have found degradation of the endothelial layer after pediatric cardiac surgery with CPB [5, 29]. Studies in adults also observed shedding of the endothelial glycocalyx and increased endothelial biomarkers after cardiac surgery [20, 21, 30, 31]. Both findings were associated with disturbed microcirculatory perfusion, as damage to the endothelial layer would delay perfusion through these vessels [32].

The decreased perfusion quality and the increased RBCv of small vessels after surgery could have been signs of hemodilution. However, changes in these microcirculatory parameters were not associated with changes in hemoglobin or hematocrit levels (data not shown). As we cannot yet quantify capillary hematocrit, it remains uncertain whether hemodilution occurred at the microcirculatory level. Decreased microcirculatory perfusion and vessel density after surgery could have also resulted from hypothermia during cardiac surgery. Previous studies have shown that hypothermia reduced microcirculatory perfusion and that perfusion recovered upon rewarming [33, 34]. As our study patients were not measured during but only after CPB and rewarming, we could not assess the effect of hypothermia on the microcirculation. Children, however, could be more susceptible to the effect of hypothermia. Larger studies have yet to conclusively demonstrate whether these pre- and postoperative disturbances are predictive of postoperative morbidity.

### RBCv, a New Parameter

RBCv was assessed for the first time in pediatric microcirculatory imaging with recently introduced automated analysis software MicroTools [3]. CHD did not affect the RBCv as RBCv did not differ between patients with and without CHD. RBCv significantly increased after cardiac surgery, mainly in patients who showed unaltered microcirculatory perfusion.

### Limitations

Some limitations need to be addressed. We have performed an observational study with a heterogeneous group of CHD. Yet, this study population is a good representation of our patient population. Age was inversely correlated with density parameters, in line with previous research [15–17]. Although age did not significantly differ between the cardiac surgery and the non-cardiac surgery group, exact age-matching was not possible. Through multivariate analysis, we were able to correct for age. Due to the young age of our study population, we were unable to perform measurements before the induction of anesthetics or after surgery when sedation was ceased. The sublingual microcirculation was possibly affected through manipulation during intubation and the presence of the endotracheal tube, the trans-esophageal echography probe, and the feeding tube. However, as baseline measurements were not possible, this effect could not be investigated. Induction of anesthesia and the type of anesthetic drugs administered could also have influenced the microcirculation [35, 36]. Also, no intraoperative measurements could be performed due to limited physical space. Whether the sublingual microcirculation is representative of other tissues could be disputed as it remains unclear how it correlates to other tissues. However, the sublingual mucosa is a relatively easily accessible area and sublingual microcirculatory disturbances are associated with worse outcomes [14].

## Conclusion

 In﻿ summary, this study has demonstrated that the presence of CHD in children is associated with disturbances of the sublingual microcirculation and the sublingual microcirculation is altered after cardiac surgery with CPB. The presence of disturbed microcirculatory perfusion could be a warning sign for altered tissue oxygenation and requires further exploration to assess the potential of microcirculatory monitoring for clinical practice.

### Supplementary Information

ESM 1

## Abbreviations

CHD: Congenital heart disease
CPB: Cardiopulmonary bypass
IDF: Incident dark field illumination
IS: Inotrope score
MFI: Microcirculatory flow index
PPV: Proportion of perfused vessels
PVD: Perfused vessel density
RACHS-1: Risk Adjustment for Congenital Heart Surgery 1
RBCv: Red blood cell velocity
SDF: Sidestream dark field imaging
SaO_2_: Arterial oxygen saturation
SpO_2_: Peripheral oxygen saturation
TVD: Total vessel density
VIS: Vasoactive-inotrope score

## Declarations

### Disclaimer

Stichting Sophia Kinderziekenhuis Fonds was in no way involved in the study design, data collection and analysis, decision to publish, or preparation of this manuscript.
