# Supplementary material for: Microcirculatory Monitoring in Children with Congenital Heart Disease Before and After Cardiac Surgery
Source: J Cardiovasc Transl Res. 2023 Jul 14;16(6):1333–42. doi: 10.1007/s12265-023-10407-4 (PMC10721654; doi:10.1007/s12265-023-10407-4)
Supplement: Supplementary file 1 — ESM 1 [file 12265_2023_10407_MOESM1_ESM.pdf]

Supplementary Information

Supplementary information belonging to article:

MICROCIRCULATORY MONITORING IN CHILDREN WITH CONGENITAL HEART DISEASE BEFORE AND AFTER CARDIAC SURGERY

Özge Erdem; Jurgen C. de Graaff (MD, PhD); Matthias P. Hilty (MD); Ulrike S. Kraemer (MD); Inge I. de Liefde (MD, PhD); Joost van Rosmalen (PhD); Can Ince (MD, PhD); Dick Tibboel (MD, PhD); Jan Willem Kuiper (MD, PhD); *Published in the* ***Journal of Cardiovascular Translational Research***

Corresponding author: Özge Erdem, o.erdem.1@erasmusmc.nl
Intensive Care and department of Pediatric Surgery, Erasmus MC University Medical Center – Sophia Children's Hospital, Rotterdam, the Netherlands

Table 1 Diagnoses of the cardiac surgery group

Diagnosis	Number of patients (n=38)	Number of cyanotic patients (n=9)
Tetralogy of Fallot	7	1
Ventricular septal defect	6	0
Atrial septal defect	5	0
Atrioventricular septal defect	3	0
Atrial septal defect and pulmonary valve stenosis	3	0
Transposition of the great arteries	2	1
Atrial septal defect and ventricular septal defect	1	0
Atrial septal defect and transposition of the great arteries	1	1
Atrial septal defect and Scimitar syndrome	1	0
Atrial septal defect, coarctation of the aorta and hypoplastic aortic arch	1	0
Ventricular septal defect, transposition of the great arteries, double inlet left ventricle, coarctation of the aorta	1	1
Hypoplastic right heart syndrome and pulmonary atresia	1	1
Hypoplastic right heart syndrome, ventricular septal defect, transposition great arteries, pulmonary valve stenosis	1	1
Hypoplastic right heart syndrome, atrial septal defect and ventricular septal defect	1	1
Hypoplastic left heart syndrome and coarctation of the aorta	1	1
Hypoplastic left heart syndrome, coarctation of the aorta, hypoplastic aortic arch, atrial septal defect	1	0
Hypoplastic left heart syndrome, aortic valve stenosis, atrial septal defect	1	1
Bicuspid aortic valve and aortic valve stenosis	1	0

Categorical data are presented as n. CHD = congenital heart disease.

Table 2 Surgeries of the study population

Group	RACHS-1 category	Surgery	n
**Cardiac surgery (n=38)**	**1**	Closure atrial septal defect	6
		Closure atrial septal defect and repair partially anomalous pulmonary venous connection	1
	**2**	Closure ventricular septal defect	7
		Total repair of tetralogy of Fallot	4
		Closure atrial septal defect and closure ventricular septal defect	1
		Glenn procedure	1
		Glenn procedure, debanding pulmonary artery, closure pulmonary valve	1
		Pulmonary valve replacement	1
		Pulmonary valvotomy and closure atrial septal defect	1
		Closure atrial septal defect and ventricular septal defect and cancel Double-chambered right ventricle	1
		Right ventricular outflow tract augmentation and pulmonary valve replacement	1
		Right ventricular outflow tract augmentation	1
		Repair of pulmonary artery stenosis and ascending aorta stenosis	1
	**3**	Correction atrioventricular septal defect	2
		Correction atrioventricular septal defect, debanding pulmonary artery, widening pulmonary artery through patch insertion	2
		Fontan procedure	1
		Aortic valve replacement	1
		Arterial switch	1
		Arterial switch and closure atrial septal defect	1
	**4**	Repair hypoplastic aortic arch and closure atrial septal defect	1
		Repair hypoplastic aortic arch, closure atrial septal defect, and correction coarctation	1
	**6**	Norwood procedure	1
**Non-cardiac surgery (n=35)**	-	Cleft surgery (lip and/or palate)	10
	-	Esophagus dilatation or esophagoscopy	2
	-	Laparoscopic fundoplication	1
	-	Laparoscopic surgery	1
	-	Excision nasal dermoid	1
	-	Excision congenital hemangioma	1
	-	Resection neurofibroma eyelid	1
	-	Resection lateral fistula in the neck and adenotonsillectomy	1
	-	Excision median neck cyst	1
	-	Cranioplasty procedures	7
	-	Fronto-orbital remodelling	1
	-	Adenotonsillectomy	3
	-	Placement bone anchored hearing aid	1
	-	Open reduction of congenital hip dislocation	2
	-	Open reduction of congenital hip dislocation and adenotonsillectomy	1
	-	Open pyeloplasty	1

RACHS-1 = Risk Adjustment for Congenital Heart Surgery 1

Table 3 Microcirculatory parameters at (T_1) Before surgery: cardiac surgery group versus non-cardiac surgery group

Variable	Cardiac surgery group (n=35)	Non-cardiac surgery group (n=35)	P-value
TVD_{all}, mm/mm^2	27.99 (25.62 - 31.31)	27.47 (24.46 – 29.40)	0.114
TVD_{small}, mm/mm^2	24.18 (22.08 - 27.31)	22.45 (20.04 – 24.93)	0.064
PPV_{all}.%	98.84 (97.99 - 99.53)	100.00 (99.44 – 100.00)	<0.001*
PPV_{small}, %	98.68 (97.70 - 99.44)	100.00 (99.32 – 100.00)	<0.001*
PVD_{all}, mm/mm^2	27.62 (25.36 - 30.71)	27.47 (24.46 – 29.67)	0.252
PVD_{small}, mm/mm^2	23.40 (21.82 - 26.71)	22.45 (19.88 – 24.93)	0.114
MFI_{all}	2.9 (2.8 - 2.9)	3.0 (3.0 – 3.0)	<0.001*
MFI_{all} < 2.6, n (%)	0 (0%)	0 (0%)	1.000
MFI_{small}	2.8 (2.5 – 2.9)	3.0 (2.9 – 3.0)	<0.001*
MFI_{small}< 2.6, n (%)	12 (34%)	0 (0%)	<0.001*
RBCv, µm/s	354.6 (332.9 – 383.0)	367.7 (356.3 – 386.7)	0.072

*Data of time point (T1) Before surgery are shown. Continuous data are presented as median (interquartile range), categorical data as n (%). The last column shows the p-values based on the Mann-Whitney U test for continuous data and the Pearson's chi-square test for categorical data. CHD = congenital heart disease; MFI=microcirculatory flow index; PPV=proportion of perfused vessels; PVD=perfused vessel density; RBCv = red blood cell velocity. TVD=total vessel density. * A p-value <0.05 was considered significant.*

Table 4 Linear regression models for density parameters: cardiac surgery group versus non-cardiac surgery group

Model	Dependent variable	Covariate	Estimate	95% CI	p-value
1	TVD_{all}, mm/mm^2	Intercept	27.32	25.81 – 28.82	<0.001*
		Group:			0.085
		Cardiac surgery group	1.35	-0.19 – 2.89	
		Non-cardiac surgery group	0 (reference)		
		Sex:			0.147
		Male	1.15	-0.40 – 2.70	
		Female	0 (reference)		
		Age, years	-0.32	-0.58 – -0.06	0.016*
2	TVD_{small}, mm/mm^2	Intercept	23.01	21.46 – 24.56	<0.001*
		Group:			0.039*
		Cardiac surgery group	1.66	0.08 – 3.24	
		Non-cardiac surgery group	0 (reference)		
		Sex:			0.080
		Male	1.42	-0.17 – 3.01	
		Female	0 (reference)		
		Age, years	-0.44	-0.71 – -0.18	0.001*
3	PVD_{all}, mm/mm^2	Intercept	27.12	25.62 – 28.61	<0.001*
		Group:			0.206
		Cardiac surgery group	0.98	-0.54 – 2.51	
		Non-cardiac surgery group	0 (reference)		
		Sex:			0.117
		Male	1.22	-0.31 – 2.76	
		Female	0 (reference)		
		Age, years	-0.29	-0.55 – -0.03	0.026*
4	PVD_{small}, mm/mm^2	Intercept	22.83	21.29 – 24.34	<0.001*
		Group:			0.102
		Cardiac surgery group	1.30	-0.26 – 2.85	
		Non-cardiac surgery group	0 (reference)		
		Sex:			0.061
		Male	1.49	-0.07 – 3.06	
		Female	0 (reference)		
		Age, years	-0.42	-0.68 – -0.16	0.002*

*Data of time point (T1) Before surgery was used. TVD = total vessel density; PVD = perfused vessel density; * = p-value < 0.05 was considered significant.*

Table 5 Perioperative demographics of the cardiac surgery group

**Variable**	
Induction of anesthetics:<br>Volatile (sevoflurane)<br>Intravenous (propofol)	 29 (76%)<br>9 (24%)
Maintenance anesthetics: Intravenous (propofol or midazolam)	38 (100%)
Surgery duration, min	173 (135 – 219)
Cardiopulmonary bypass duration, min	101 (58 – 144)
Aortic cross-clamping	35 (92%)
Aortic cross-clamping time, min	66 (37 – 92)
Hypothermia (<35 °C)	32 (84%)
Hypothermia, °C	32 (30-32)
Deep hypothermic circulatory arrest (18-20 °C)	3 (8%)
Deep hypothermic circulatory arrest time, min	51 (17 – 64)
Antegrade cerebral perfusion	3 (8%)
Antegrade cerebral perfusion time, min	43 (41 – 47)
Priming solution included erythrocytes	22 (58%)
Blood loss during surgery, ml/kg	125.5 (31.5 – 215.3)
Unfractionated heparin, IU/kg	646 (543 – 820)
Protamine, mg/kg	5.0 (4.4 – 5.4)
Intraoperative transfusion including CPB priming, ml/kg:<br>Blood products (n=37)<br>Plasma products (n=38)<br>Colloids (n=36)<br>Crystalloids (n=20)	 125.5 (31.5 – 215.3)<br>44.8 (30.0 – 65.9)<br>4.9 (2.7 – 6.9)<br>1.5 (0.0 -7.3)
Postoperative diuretics	29 (76%)
Second surgical look	2 (5%)
Total duration mechanical ventilation, hrs	10 (7 – 22)
Postoperative duration mechanical ventilation, hrs	7 (4 – 19)
Low cardiac output syndrome, n (%)	7 (18%)
ICU duration of stay, days	1 (1 – 4)
Hospital duration of stay, days	7 (6 – 11)
Survival	38 (100%)

Continuous data are presented as median (interquartile range), categorical data as n (%). NA = not applicable.

Table 6 Perioperative microcirculatory parameters of the cardiac surgery group

Variable	(T_1) Before surgery	(T_2) After wound closure	*P-value*	(T_3) 1h after surgery	(T_4) 4h after surgery	(T_5) 6h after surgery	P-value
Patients	38	38	NA	38	38	38	-
Intubated and sedated	38	38	NA	31	21	7	-
Measurements	35	34	NA	31	21	7	-
TVD_{all}, mm/mm^2	27.99 (25.62 - 31.31)	26.34 (25.21 - 29.57)	0.050	27.23 (24.61 - 30.97)	26.86 (24.84 - 30.68)	27.50 (25.30 - 29.34)	0.271
TVD_{small}, mm/mm^2	24.18 (22.08 - 27.31)	22.26 (21.22 - 25.45)	0.112	23.17 (21.15 - 26.94)	23.45 (21.30 - 26.67)	24.98 (21.69 - 27.28)	0.661
PPV_{all} ,%	98.84 (97.99 - 99.53)	98.26 (96.61 - 99.07)	0.352	97.39 (95.96 - 99.21)	97.95 (95.98 - 99.45)	98.84 (96.45 - 99.49)	-
PPV_{small}, %	98.68 (97.70 - 99.44)	97.93 (96.14 - 98.95)	0.327	97.23 (95.28 - 99.17)	97.69 (95.07 - 99.32)	98.59 (96.00 - 99.48)	-
PVD_{all}, mm/mm^2	27.62 (25.36 - 30.71)	25.84 (23.94 - 29.23)	0.046*	26.41 (24.11 - 30.82)	26.27 (23.69 - 30.16)	27.40 (24.61 - 29.12)	0.288
PVD_{small}, mm/mm^2	23.40 (21.82 - 26.71)	21.98 (20.65 - 25.14)	0.088	22.73 (19.98 - 25.11)	22.66 (20.28 - 26.14)	23.85 (21.01 - 27.06)	0.587
MFI_{all}	2.9 (2.8 - 2.9)	2.8 (2.7 - 2.9)	0.218	2.8 (2.6 - 2.9)	2.8 (2.5 – 3.0)	2.7 (2.6 - 2.9)	-
MFI_{all} < 2.6, n (%)	0	3 (9%)	0.114	9 (29%)	6 (29%)	1 (14%)	0.041*
MFI_{small}	2.8 (2.5 – 2.9)	2.5 (2.3 – 2.9)	0.038*	2.5 (2.3 – 2.8)	2.5 (2.3 – 3.0)	2.3 (2.1 – 2.8)	-
MFI_{small}< 2.6, n (%)	12 (34%)	19 (56%)	0.071	20 (65%)	11 (52%)	4 (57%)	0.008*
RBCv, µm/s	354.6 (332.9 – 383.0)	369.7 (348.3 – 393.6)	0.032*	360.6 (348.5 – 382.3)	354.9 (333.9 – 399.3)	339.2 (332.8 – 339.2)	-

Continuous data are presented as median (interquartile range), categorical data as n (%).The fourth column shows the p-values based on the Wilcoxon Signed Ranks test for continuous data and the Pearson Chi-Square test for categorical data to compare (T_1) and (T_2). The last column shows the p-values based on the linear mixed models for normally distributed continuous variables TVD and PVD corrected for age and sex and GEE logistic regression models for categorical variable MFI to compare all time points. CHD = congenital heart disease; MFI = microcirculatory flow index; NA = not applicable; PPV = proportion of perfused vessels; PVD=perfused vessel density; RBCv = red blood cell velocity. TVD = total vessel density.

** A p-value < 0.05 was considered statistically significant.*

Table 7 Linear mixed models of density parameters: (T_1) Before surgery versus (T_2) After wound closure

Model	Dependent variable	Covariate	Estimate	95% CI	p-value
1	**TVD_{all}**	Intercept	26.82	25.20 – 28.45	<0.001*
		Time point:<br>(T_1) Before surgery<br>(T_2) After wound closure	 1.61<br>0 (reference)	 0.15 – 3.07	0.032*
		Sex:<br>Male<br>Female	 1.12<br>0 (reference)	 -0.70 – 2.94	0.219
		Age, years	-0.22	-0.44 – -0.01	0.049*
2	**TVD_{small}**	Intercept	23.22	21.59 – 24.84	<0.001*
		Time point:<br>(T_1) Before surgery<br>(T_2) After wound closure	 1.17<br>0 (reference)	 -0.19 – 2.52	0.088
		Sex:<br>Male<br>Female	 1.51<br>0 (reference)	-0.35 – 3.36	0.108
		Age, years	-0.36	-0.58 – -0.14	0.002*
3	**PVD_{all}**	Intercept	26.11	24.52 – 27.71	<0.001*
		Time point:<br>(T_1) Before surgery<br>(T_2) After wound closure	 1.75<br>0 (reference)	 0.24 – 3.26<br>-	0.025*
		Sex:<br>Male<br>Female	 1.29<br>0 (reference)	-0.48 – 3.06	0.148
		Age, years	-0.21	-0.43 – -0.01	0.054
4	**PVD_{small}**	Intercept	22.52	20.93 – 24.11	<0.001*
		Time point:<br>(T_1) Before surgery<br>(T_2) After wound closure	 1.30<br>0 (reference)	 -0.06 – 2.66<br>-	0.060
		Sex:<br>Male<br>Female	 1.68<br>0 (reference)	 -0.12 – 3.48	0.067
		Age, years	-0.35	-0.57 – -0.13	0.002*

*Data of the cardiac surgery group on time points (T_1) Before surgery and (T_2) After wound closure were used. TVD = total vessel density; PVD = perfused vessel density; * = A p-value < 0.05 was considered significant.*
